# Socioeconomic and Comorbid Factors Affecting Mortality and Length of Stay in COVID-19 Patients

**DOI:** 10.7759/cureus.30224

**Published:** 2022-10-12

**Authors:** Adam Delora, Ashlynn Mills, David Jacobson, Brendon Cornett, William F Peacock, Anita Datta, Shane P Jenks

**Affiliations:** 1 Emergency Medicine, HCA Houston Healthcare Kingwood/University of Houston College of Medicine, Kingwood, USA; 2 Emergency Medicine, University of Houston College of Medicine, Houston, USA; 3 Graduate Medical Education, HCA Healthcare, Denver, USA; 4 Emergency Medicine, Baylor College of Medicine, Houston, USA

**Keywords:** access to healthcare and health outcomes of vulnerable populations, healthcare inequality, racial and ethnic disparities, social determinants of health (sdoh), covid-19

## Abstract

Background

The severe acute respiratory syndrome coronavirus 2 (SARS-CoV-2) pandemic exposed and exacerbated health disparities between socioeconomic groups. Our purpose was to determine if age, sex, race, insurance, and comorbidities predicted patients' length of stay (LOS) in the hospital and in-hospital mortality in patients diagnosed with coronavirus disease 2019 (COVID-19) during the early pandemic.

Methods

Utilizing retrospective, secondarily sourced electronic health record (EHR) data for patients who tested positive for COVID-19 from HCA Healthcare facilities, predictors of LOS and in-hospital mortality were assessed using regression. LOS and in-hospital mortality were assessed using logistic regression and negative binomial regression, respectively. All models included age, insurance status, and sex, while additional covariates were selected using the least absolute shrinkage and selection operator (LASSO) regression. LOS data were presented as incidence rate ratios (IRR), and in-hospital mortality was presented as odds ratios (OR), followed by their 95% confidence intervals (CI).

Results

There were 111,849 qualifying patient records from March 1, 2020, to August 23, 2020. After excluding those with missing data (n = 7), without clinically confirmed COVID-19 (n = 27,225), and those from a carceral environment (n = 1,861), there were 84,624 eligible patients. Compared to the population of the United States of America, our COVID-19 cohort had a larger proportion of African American* *patients (23.17% versus 13.4%). The African American patients were more likely to have private insurance providers (28.52% versus 23.68%) and shorter LOS (IRR = 0.88, 95% CI = 0.86-0.90) than the White patient cohort. In addition, the African American versus White patients did not have increased odds (OR = 0.98, 95% CI = 0.96-1.00) of in-hospital mortality. Patients on Medicaid (OR = 1.04, 95% CI = 1.01-1.07) and self-pay (OR = 1.07, 95% CI = 1.00-1.14, noninclusive endpoints) had higher in-hospital mortality than private insurance. Several comorbidities were predictive of an increased LOS, including anxiety (IRR = 1.94, 95% CI = 1.87-2.01) and sedative abuse (IRR = 2.07, 95% CI = 1.63-2.64).

Conclusions

Race was not associated with increased LOS or in-hospital mortality in patients with COVID-19 infections during the early pandemic. Insurance type, psychiatric comorbidities, and medical comorbidities significantly impacted outcomes in patients with COVID-19. This research and future research in the field should help to determine rational public policies to help mitigate the risk of diseases and their impact on future pandemics.

## Introduction

It is rare for a pandemic to affect society uniformly. In the 14th century, the Black Death disproportionately affected the poorest in society. In medieval Europe, the bubonic plague resulted in a greater proportion of deaths in the lower-income working class [1]. In December 2019, an outbreak of coronavirus disease 2019 (COVID-19) caused by the severe acute respiratory syndrome coronavirus 2 (SARS-CoV-2) virus spread globally [2]. Several articles have been published on health disparities among different populations with COVID-19 and have reported poorer outcomes in minority communities, including persons of American Indian, African American, and Hispanic descent [2-12]. Early in the pandemic, several papers were published in which health disparities were examined in African Americans, and several factors were hypothesized to explain the greater risk of COVID-19 infection among African Americans. Among these factors were densely populated housing, a greater burden of chronic disease, limited access to health care, higher poverty rates, and a higher likelihood of employment as essential workers [13,14]. However, while systemic inequality has been conclusively shown to contribute to morbidity and mortality in other infectious diseases, additional work is needed to examine the interaction between different social determinants of health (SDoH) and COVID-19 [15-21]. Systemic social inequality and discrepancies in socioeconomic status (SES) contribute to a higher incidence of asthma, cerebrovascular disease (CVD), hypertension, chronic kidney disease (CKD), and obesity in segments of the general population [22]. These social disparities point to a national need for patient data analysis to describe and substantiate the various disparities and their associated factors.

In data extracted from COVID-Net and National Center for Health Statistics (NCHS), the Centers for Disease Control and Prevention (CDC) reported 2.8x, 2.2x, and 2.1x higher hospitalization rates for American Indian, African American, and Hispanic patients, respectively, when compared to non-Hispanic White patients. Death rates were also 2.1x, 1.7x, and 1.8x higher in these groups, respectively [3]. However, these data do not take into account associated comorbidities that affect outcomes in COVID-19. The CDC investigated several factors that may affect mortality in COVID-19, including long-standing systemic health and social inequities. Unfortunately, because much of the data are missing in CDC populations, with roughly 40-50% of race and ethnicity data unaccounted for, definitive conclusions about the data cannot be made. Also, age was not accounted for, and comorbidity corrections were not made in the CDC data [3,23].

The payer type can serve as a proxy for SES and give insight into access to health care. According to the Census Bureau, in 2019, 87.6% of people with an income-to-poverty ratio at or above 400% of poverty had private insurance coverage. People of lower SES had lower rates of private insurance coverage, with 60.1% private insurance for those with incomes 100-399% of poverty and only 26.6% for those with incomes below poverty. In contrast, public coverage was the most prevalent for the population in poverty (65.2%) and least prevalent for the population with income-to-poverty ratios at or above 400% of poverty (21.9%). Medicaid requires that an applicant is below a certain income level. Also, under the Affordable Care Act, individual states can expand Medicaid eligibility to people whose income-to-poverty ratio falls under a particular threshold. The extent of poverty required to be eligible for Medicaid varies marginally depending on whether that state expanded Medicaid eligibility [24].

Understanding the effects of SDoH and their interactions with comorbidities can facilitate rational public programs and contribute to our knowledge of health disparities in the context of the COVID-19 pandemic and possibly future pandemics. High-quality data on socioeconomic factors are needed to identify groups that are most likely to have poor outcomes, which will have important implications for developing public health measures [25]. HCA Healthcare is the largest health system in the United States and thus provides a suitable database to evaluate SDoH in relation to SARS-CoV-2 infection-associated outcomes. Given that HCA Healthcare is a private hospital system, there are fewer gaps in data given that data are more uniformly gathered throughout the network.

This study aimed to examine the interaction of comorbidities, access to insurance, and demographics and how they predict outcomes in an early and modern pandemic. Understanding the effects of SDoH and their interactions with comorbidities can facilitate responsive public programs, contribute to our knowledge of health disparities in the context of the COVID-19 pandemic, and provide lessons that can be used in managing future pandemics. We endeavored to determine the impact of selected disparities and comorbidities on the length of stay (LOS) and in-hospital mortality in patients diagnosed with COVID-19. A previous version of this article was previously posted to the medRxiv preprint server on August 28, 2021.

## Materials and methods

Data were obtained from 182 different HCA facilities treating COVID-19 patients. Patients were included only if they tested positive for COVID-19 with the polymerase chain reaction (PCR) or antigen test during the period from March 1, 2020, to August 23, 2020. The data were de-identified and then given to our team. Each patient had a unique identifier along with their age, insurance status, sex, self-identified race, comorbidities, LOS, in-hospital mortality, and type of insurance. Patients were excluded from analysis if they did not have a clinical diagnosis of COVID-19 based on billing and coding data. They were also excluded if they tested negative for COVID-19, had missing data, or were transferred to an HCA Healthcare facility from a carceral environment. The incarcerated population was deemed not representative of the population of interest for this analysis, but further research in this cohort may be performed in future studies. The Elixhauser Comorbidity Index (ECI) was calculated for each patient. The index is a comorbidity measure that includes comorbidities associated with substantial increases in length of stay, hospital charges, and mortality for heterogeneous and homogeneous disease groups [26]. Descriptive statistics, like percentages, medians, and interquartile ranges (IQRs) for independent and dependent variables, were calculated to provide contextual information about the study participants' clinical and demographic characteristics.

We created regression models with dependent variables of LOS and in-hospital mortality. Age, insurance status, sex, and ECI were included in all models as these were variables of high clinical importance within the study, while additional comorbidity covariates were selected using the least absolute shrinkage and selection operator (LASSO) regression. Logistic regression utilizing Firth's logistic regression with added covariate (FLAC) model for rare events was used to assess in-hospital mortality. Firth's logistic method is useful in reducing the bias when there is a strong imbalance in the outcome [27]. LOS values were given to us in days as whole numbers. We performed a negative binomial regression with these discrete values to assess the LOS. All effect sizes are reported as odds ratios (OR) or incidence rate ratios (IRR) with 95% confidence intervals (CI).

## Results

There were 111,849 patient records initially selected. A total of 27,225 patients were excluded because 25,357 did not have a clinical diagnosis of COVID-19 by a clinician, seven were missing demographic data (gender, race, etc.), and 1,861 patients were remanded to law enforcement or were transferred from a carceral environment. The remaining 84,624 patients constituted the analytic cohort. Demographics, insurance type, and hospital stay characteristics binned by mortality are presented in Table 1.

**Table 1:** Demographics and hospital stay characteristics by mortality This table shows mortality binned on certain demographic data and insurance types. We included raw counts and percentages.

	Expired (N = 8,159)	Survived (N = 76,465)	Total (N = 84,624)
Age			
Median (interquartile range)	76 (65-84)	50 (35-65)	52 (36-68)
Range	18-91	18-91	18-91
Gender			
Female	3,625 (44.43%)	40,698 (53.22%)	44,323 (52.38%)
Male	4,534 (55.57%)	35,767 (46.78%)	40,301 (47.62%)
Race			
Asian	200 (2.45%)	1,548 (2.02%)	1,748 (2.07%)
African American	1,284 (15.74%)	18,326 (23.97%)	19,610 (23.17%)
Hispanic	16 (0.20%)	166 (0.22%)	182 (0.22%)
Multiracial/Other	1,561 (19.13%)	19,812 (25.91%)	21,373 (25.26%)
American Indian	17 (0.21%)	150 (0.20%)	167 (0.20%)
White	5,081 (62.27%)	36,463 (47.69%)	41,544 (49.09%)
Length of stay			
Median (interquartile range)	9 (4-16)	1 (0-4)	1 (0-6)
Range	0-170	0-196	0-196
Rehabilitation facility	0 (0.00%)	6,231 (8.15%)	6,231 (7.36%)
Payment type			
Charity	15 (0.18%)	427 (0.56%)	442 (0.52%)
Government	742 (9.09%)	18,932 (24.76%)	19,674 (23.25%)
Standard insurance	665 (8.15%)	19,608 (25.64%)	20,273 (23.96%)
Medicaid	521 (6.39%)	10,114 (13.23%)	10,635 (12.57%)
Medicare	5,987 (73.38%)	21,303 (27.86%)	27,290 (32.25%)
Other	170 (2.08%)	4,864 (6.36%)	5,034 (5.95%)
Self-pay	59 (0.72%)	1,217 (1.59%)	1,276 (1.51%)
Admission source			
From home	6,612 (81.04%)	72,643 (95.00%)	79,255 (93.66%)
Hospital transfer	1,547 (18.96%)	3,822 (5.00%)	5,369 (6.34%)
Encounter type			
Emergency	185 (2.27%)	38,818 (50.77%)	39,003 (46.09%)
Inpatient	7,973 (97.72%)	35,072 (45.87%)	43,045 (50.87%)
Observation	1 (0.01%)	2,084 (2.73%)	2,085 (2.46%)
Same-day surgery	0 (0.00%)	491 (0.64%)	491 (0.58%)

Demographics and hospital stay characteristics by race are presented in Table 2. Our population self-identified as 52.38% female and was 49.09% White, 23.17% African American, 25.26% multiracial, 0.20% American Indian, and 0.22% Hispanic. Our African American population was larger than the general population of the USA, i.e., 23.17% vs. 13.4%, respectively. The percentage of our population who considered themselves multiracial or did not fit into the predefined groups was also much larger than the general population (25.26% vs. 2.6%) [28].

**Table 2:** Demographics and hospital stay characteristics by race This table shows demographics like age and insurance and the comorbidities we evaluated binned by race. We included raw counts and percentages.

	Asian (N = 1,748)	African American (N = 19,610)	Hispanic (N = 182)	Multiracial/other (N = 21,373)	American Indian (N = 167)	White (N = 41,544)	Total (N = 84,624)
Age							
Median (interquartile range)	57 (43-71)	48 (33-63)	50 (37-62)	47 (34-60)	49 (36-65)	58 (41-74)	52 (36-68)
Range	18-91	18-91	19-91	18-91	18-91	18-91	18-91
Gender							
Female	873 (49.94%)	11,451 (58.39%)	117 (64.29%)	10,482 (49.04%)	91 (54.49%)	21,309 (51.29%)	44,323 (52.38%)
Male	875 (50.06%)	8,159 (41.61%)	65 (35.71%)	10,891 (50.96%)	76 (45.51%)	20,235 (48.71%)	40,301 (47.62%)
Length of stay							
Median (interquartile range)	2 (0-7)	1 (0-5)	1 (0-4)	1 (0-5)	2 (0-7)	2 (0-6)	1 (0-6)
Range	0-86	0-98	0-39	0-85	0-36	0-196	0-196
Discharge category							
Expired	200 (11.44%)	1,284 (6.55%)	16 (8.79%)	1,561 (7.30%)	17 (10.18%)	5,081 (12.23%)	8,159 (9.64%)
Home	1,364 (78.03%)	16,668 (85.00%)	157 (86.26%)	18,327 (85.75%)	132 (79.04%)	30,773 (74.07%)	67,421 (79.67%)
Hospital	49 (2.80%)	513 (2.62%)	5 (2.75%)	700 (3.28%)	5 (2.99%)	1,541 (3.71%)	2,813 (3.32%)
Rehabilitation facility	135 (7.72%)	1,145 (5.84%)	4 (2.20%)	785 (3.67%)	13 (7.78%)	4,149 (9.99%)	6,231 (7.36%)
Payment type							
Charity	0 (0.00%)	89 (0.45%)	2 (1.10%)	152 (0.71%)	1 (0.60%)	198 (0.48%)	442 (0.52%)
Government	182 (10.41%)	3,871 (19.74%)	69 (37.91%)	8,205 (38.39%)	40 (23.95%)	7,307 (17.59%)	19,674 (23.25%)
Standard insurance	598 (34.21%)	5,593 (28.52%)	33 (18.13%)	4,177 (19.54%)	36 (21.56%)	9,836 (23.68%)	20,273 (23.96%)
Medicaid	222 (12.70%)	3,439 (17.54%)	25 (13.74%)	3,067 (14.35%)	32 (19.16%)	3,850 (9.27%)	10,635 (12.57%)
Medicare	547 (31.29%)	5,490 (28.00%)	42 (23.08%)	4,043 (18.92%)	54 (32.34%)	17,114 (41.19%)	27,290 (32.25%)
Other	182 (10.41%)	877 (4.47%)	7 (3.85%)	1,207 (5.65%)	3 (1.80%)	2,758 (6.64%)	5,034 (5.95%)
Self-pay	17 (0.97%)	251 (1.28%)	4 (2.20%)	522 (2.44%)	1 (0.60%)	481 (1.16%)	1,276 (1.51%)
Admission source							
From home	1,648 (94.28%)	18,666 (95.19%)	174 (95.60%)	20,331 (95.12%)	163 (97.60%)	38,273 (92.13%)	79,255 (93.66%)
Hospital transfer	100 (5.72%)	944 (4.81%)	8 (4.40%)	1,042 (4.88%)	4 (2.40%)	3,271 (7.87%)	5,369 (6.34%)
Encounter type							
Emergency	707 (40.45%)	10,437 (53.22%)	98 (53.85%)	10,694 (50.04%)	64 (38.32%)	17,003 (40.93%)	39,003 (46.09%)
Inpatient	986 (56.41%)	8,826 (45.01%)	80 (43.96%)	10,078 (47.15%)	98 (58.68%)	22,977 (55.31%)	43,045 (50.87%)
Observation	48 (2.75%)	284 (1.45%)	4 (2.20%)	504 (2.36%)	2 (1.20%)	1,243 (2.99%)	2,085 (2.46%)
Same-day surgery	7 (0.40%)	63 (0.32%)	0 (0.00%)	97 (0.45%)	3 (1.80%)	321 (0.77%)	491 (0.58%)

Comorbidity summary statistics by mortality are presented in Table 3. The three most common conditions were hypertension (n = 39,154, 46.27%), diabetes (n = 24,352, 28.78%), and sepsis (n = 17,122, 20.23%). The payer mix included Medicare (n = 27,290, 32.25%), Medicaid (n = 10,635, 12.57%), private insurance (n = 20,273, 23.96%), and government insurance through the state or through the healthcare exchange (n = 19,674, 23.25%). Few patients reported charity pay (0.52%) or other payer sources that did not fall into the above categories (7.46%).

**Table 3:** Comorbidities by mortality This table shows selected comorbidities and the Elixhauser Comorbidity Index median and range by mortality. We included raw counts and percentages.

	Expired (N = 8,159)	Survived (N = 76,465)	Total (N = 84,624)
Elixhauser Comorbidity Index			
Median (interquartile range)	3 (2-5)	1 (0-2)	1 (0-2)
Range	0-21	0-19	0-21
Alcohol abuse	220 (2.70%)	1,016 (1.33%)	1,236 (1.46%)
Anxiety	1,308 (16.03%)	4,848 (6.34%)	6,156 (7.27%)
Bipolar disorder	137 (1.68%)	743 (0.97%)	880 (1.04%)
Cerebrovascular disease	551 (6.75%)	1,109 (1.45%)	1,660 (1.96%)
Chronic kidney disease	3,119 (38.23%)	6,541 (8.55%)	9,660 (11.42%)
Depression	1,006 (12.33%)	4,254 (5.56%)	5,260 (6.22%)
Diabetes	4,227 (51.81%)	20,125 (26.32%)	24,352 (28.78%)
Heart failure	2,651 (32.49%)	5,504 (7.20%)	8,155 (9.64%)
Hypotension	651 (7.98%)	1,375 (1.80%)	2,026 (2.39%)
Long-term opioid use	231 (2.83%)	925 (1.21%)	1,156 (1.37%)
Myocardial infarction	1,202 (14.73%)	1,268 (1.66%)	2,470 (2.92%)
Opioid abuse	79 (0.97%)	255 (0.33%)	334 (0.39%)
Pregnancy	6 (0.07%)	2,598 (3.40%)	2,604 (3.08%)
Post-traumatic stress disorder	65 (0.80%)	430 (0.56%)	495 (0.58%)
Pulmonary embolism	225 (2.76%)	643 (0.84%)	868 (1.03%)
Schizophrenia	211 (2.59%)	859 (1.12%)	1,070 (1.26%)
Sedative abuse	50 (0.61%)	65 (0.09%)	115 (0.14%)
Sepsis	5,564 (68.19%)	11,558 (15.12%)	17,122 (20.23%)
Suicidal	15 (0.18%)	255 (0.33%)	270 (0.32%)
Tobacco abuse	423 (5.18%)	5,608 (7.33%)	6,031 (7.13%)
Alcohol abuse	220 (2.70%)	1,016 (1.33%)	1,236 (1.46%)

Table 4 stratifies comorbidities by race. Based on the Census Bureau data, White patients were more likely to have private insurance [28]. In our study, African American patients were more likely to have private insurance than the White population (28.52% vs. 23.68%).

**Table 4:** Comorbidities of interest by race This table shows selected comorbidities and the Elixhauser Comorbidity median and range binned on race. We included raw values and percentages.

	Asian (N = 1,748)	African American (N = 19,610)	Hispanic (N = 182)	Multiracial/other (N = 21,373)	American Indian (N = 167)	White (N = 41,544)	Total (N = 84,624)
Elixhauser Comorbidity Index							
Median (interquartile range)	1 (0-2)	1 (0-2)	1 (0-2)	0 (0-2)	1 (0-2)	1 (0-2)	1 (0-2)
Range	0-12	0-19	0-7	0-17	0-13	0-21	0-21
Alcohol abuse	10 (0.57%)	189 (0.96%)	2 (1.10%)	291 (1.36%)	3 (1.80%)	741 (1.78%)	1,236 (1.46%)
Anxiety	82 (4.69%)	869 (4.43%)	19 (10.44%)	1,121 (5.24%)	15 (8.98%)	4,050 (9.75%)	6,156 (7.27%)
Bipolar disorder	5 (0.29%)	188 (0.96%)	1 (0.55%)	97 (0.45%)	3 (1.80%)	586 (1.41%)	880 (1.04%)
Cerebrovascular disease	32 (1.83%)	310 (1.58%)	1 (0.55%)	222 (1.04%)	6 (3.59%)	1,089 (2.62%)	1,660 (1.96%)
Depression	92 (5.26%)	764 (3.90%)	8 (4.40%)	709 (3.32%)	12 (7.19%)	3,675 (8.85%)	5,260 (6.22%)
Heart failure	138 (7.89%)	1,902 (9.70%)	11 (6.04%)	1,144 (5.35%)	23 (13.77%)	4,937 (11.88%)	8,155 (9.64%)
Hypotension	53 (3.03%)	383 (1.95%)	1 (0.55%)	368 (1.72%)	6 (3.59%)	1,215 (2.92%)	2,026 (2.39%)
Long-term opioid use	14 (0.80%)	163 (0.83%)	1 (0.55%)	275 (1.29%)	4 (2.40%)	699 (1.68%)	1,156 (1.37%)
Myocardial infarction	57 (3.26%)	459 (2.34%)	3 (1.65%)	486 (2.27%)	8 (4.79%)	1,457 (3.51%)	2,470 (2.92%)
Opioid abuse	3 (0.17%)	48 (0.24%)	0 (0.00%)	45 (0.21%)	3 (1.80%)	235 (0.57%)	334 (0.39%)
Pregnancy	38 (2.17%)	544 (2.77%)	7 (3.85%)	953 (4.46%)	3 (1.80%)	1,059 (2.55%)	2,604 (3.08%)
Pulmonary embolism	18 (1.03%)	259 (1.32%)	1 (0.55%)	132 (0.62%)	3 (1.80%)	455 (1.10%)	868 (1.03%)
Sedative abuse	5 (0.29%)	12 (0.06%)	0 (0.00%)	29 (0.14%)	1 (0.60%)	68 (0.16%)	115 (0.14%)
Sepsis	467 (26.72%)	3,428 (17.48%)	32 (17.58%)	4,210 (19.70%)	34 (20.36%)	8,951 (21.55%)	17,122 (20.23%)
Tobacco abuse	70 (4.00%)	1,650 (8.41%)	9 (4.95%)	1,275 (5.97%)	22 (13.17%)	3,005 (7.23%)	6,031 (7.13%)

Our primary outcomes of interest were LOS and in-hospital mortality. The results for the LOS using negative binomial regression are shown in Table 5 as IRRs.

**Table 5:** Negative binomial regression – length of stay (in days) These are the results of the negative binomial regression. Ratios are presented in days.

	Incidence rate ratio	95% confidence interval
Age	1.02	(1.02-1.02)
Sepsis	3.1	(3.03-3.18)
Elixhauser Comorbidity Index	1.22	(1.21-1.23)
Charity versus private insurance	1.9	(1.66-2.16)
Government versus private insurance	0.81	(0.78-0.83)
Medicaid versus private insurance	1.19	(1.15-1.23)
Medicare versus private insurance	0.94	(0.91-0.97)
Other insurance versus private insurance	0.88	(0.84-0.92)
Self-pay versus private insurance	0.74	(0.68-0.81)
Female versus male	0.83	(0.81-0.85)
Asian versus White	1	(0.93-1.07)
African American versus White	0.88	(0.86-0.90)
Hispanic versus White	1.07	(0.87-1.33)
Multiracial/other versus White	1.17	(1.14-1.20)
American Indian versus White	1.14	(0.92-1.42)
Anxiety	1.94	(1.87-2.01)
Cerebrovascular disease	1.07	(1.00-1.15)
Chronic kidney disease	0.86	(0.82-0.89)
Diabetes	1.11	(1.08-1.15)
Heart failure	0.83	(0.80-0.87)
Hypertension	1.23	(1.19-1.26)
Myocardial infarction	1.09	(1.03-1.15)
Pulmonary embolism	1.88	(1.72-2.06)
Sedative abuse	2.07	(1.63-2.64)

Table 6 contains logistic regression for in-hospital mortality with results shown as ORs.

**Table 6:** Logistic regression – in-hospital mortality This table shows the results of logistic regression with demographic factors and comorbidities as independent factors and in-hospital mortality as the dependent factor. We present an odds ratio and a 95% confidence interval.

	Odds ratio	95% confidence interval
Length of stay	1.01	(1.01-1.01)
Age	1	(1.00-1.01)
Sepsis	1.29	(1.26-1.32)
Elixhauser Comorbidity Index	1.02	(1.01-1.03)
Charity versus private insurance	0.99	(0.88-1.11)
Government versus private insurance	1.03	(1.01-1.06)
Medicaid versus private insurance	1.04	(1.01-1.07)
Medicare versus private insurance	1.03	(1.00-1.06)
Other versus private insurance	0.99	(0.95-1.03)
Self-pay versus private insurance	1.07	(1.00-1.14)
Female versus male	1	(0.98-1.01)
Asian versus White	0.99	(0.93-1.05)
African American versus White	0.98	(0.96-1.00)
Hispanic versus White	1.01	(0.85-1.20)
Multiracial/other versus White	0.99	(0.97-1.01)
American Indian versus White	0.99	(0.82-1.18)
Anxiety	1.03	(0.99-1.06)
Cerebrovascular disease	1.07	(1.01-1.14)
Chronic Kidney disease	1.1	(1.06-1.14)
Diabetes	0.97	(0.94-0.99)
Heart failure	1.07	(1.03-1.11)
Hypertension	0.94	(0.92-0.97)
Myocardial infarction	1.3	(1.24-1.37)
Pulmonary embolism	1.06	(0.98-1.15)
Sedative abuse	1.21	(0.97-1.49)

In our population, African American patients, as compared to White patients, had a lower incidence rate for the LOS (IRR = 0.88, 95% CI = 0.86-0.90), but similar odds of in-hospital mortality (OR = 0.98, 95% CI = 0.96-1.00). Our analysis revealed that for every one-year increase in age, the LOS was increased by 1.02 times (or 2%). Patients on Medicaid were more likely to have a longer LOS (IRR = 1.19, 95% CI = 1.15-1.23), and patients who were self-pay were predicted to have a shorter LOS (IRR = 0.74, 95% CI = 0.68-0.81) but had higher odds of in-hospital mortality (OR = 1.07, 95% CI = 1.00-1.14, noninclusive endpoints). In contrast, patients with Medicare were more likely to have a shorter LOS (IRR = 0.94, 95% CI = 0.91-0.97) and marginally increased odds of in-hospital mortality (OR = 1.03, 95% CI = 1.00-1.06). Females had shorter hospital stays (IRR = 0.83, 95% CI = 0.81-0.85) and had similar odds of in-hospital mortality compared to males (OR = 1.00, 95% CI = 0.98-1.01).

Patients with anxiety were more likely to have an increased LOS (IRR = 1.94, 95% CI = 1.87-2.01) with similar odds of in-hospital mortality (OR = 1.03, 95% CI = 0.99-1.06) to those without an anxiety diagnosis. Increased LOS and in-hospital mortality were more likely in patients with cerebrovascular disease, chronic kidney disease, myocardial infarction, and sepsis. Patients diagnosed with sedative abuse also had an increased LOS (IRR = 2.07, 95% CI = 1.63-2.64). We also included the ECI and found the LOS (IRR = 1.22, 95% CI = 1.21-1.23) and odds of in-hospital mortality (OR = 1.02, 95% CI = 1.01-1.03) increased for greater values on the ECI scale.

## Discussion

We quantitatively assessed the relationship between several SDoH and comorbidities in a COVID-19 cohort to evaluate their impact on LOS and in-hospital mortality. Payer type and comorbidities were better predictors than race for LOS and mortality in patients with COVID-19.

Healthy life expectancy and mortality rates are disproportionate between the richest and the poorest in society [1]. Attempts to use census data on poverty, household crowding, racial composition, and segregation to analyze COVID-19 data down to the level of the ZIP code area can be difficult with missing data. In addition, healthcare data and outcomes that are lacking complementary data related to SDoH may cause difficulty in making the correct conclusions regarding predictors in data [12]. SDoH were not recorded well in the early pandemic. While many early studies found a strong relationship between race and population-level mortality in COVID-19, there was substantial demographic data missing [14]. The quality of data reporting varied across states as well. There was also significant variation in the definition of mortality related to COVID-19 [4]. In the CDC data, as much as half of the demographic data were missing when calculating age-adjusted mortality rates [3]. Early data also indicated a link between comorbidities and age with outcomes in COVID-19 [23]. SDoH includes many factors like comorbidities, insurance, age, and race that all interact to determine health outcomes [9]. A more uniform dataset with additional data about comorbidities, race, and insurance was needed. To add to the existing data and look for lessons in managing patients in an early pandemic, we took enterprise-level data from HCA Healthcare at all facilities across the United States to help determine predictors of poor outcomes.

Our African American patients were more likely to have private insurance than White patients (28.52% vs. 23.68%). In our population, African American patients had a lower IRR for LOS than White patients, but African American patients had similar odds of in-hospital mortality. The White patient population had a slightly higher median ECI and was more likely to have Medicare. They also had a higher median age (58) than African American patients (48), which likely accounts for the increased number of White patients with Medicare. In fact, self-identifying as African American, Asian, Hispanic, American Indian, or multiracial/other was not a predictor of in-hospital mortality.

Insurance status was previously reported to have a strong association with income and access to care [24]. Having private insurance predicted decreased in-hospital mortality regardless of race in COVID-19 infections. These findings would indicate that genetic differences between races likely are not the cause of racial disparities in COVID-19. Rather, the incidence of comorbidities and other social determinants of health like insurance status, are more likely predictive of differences observed in other studies.

We found that age was associated with an increased LOS, as were sepsis, cerebrovascular disease, and myocardial infarction. Further, the ECI demonstrated that an increase in the comorbidity score was an important factor in predicting LOS and mortality in patients with COVID-19. Our findings both support and supplement much of the CDC’s published findings [3]. Timely identification of comorbidities is helpful for triaging COVID-19 patients when they arrive at the hospital and is also a significant predictor of mortality outside the setting of COVID-19.

The IRR for the LOS was substantially higher in patients with anxiety, but there was no statistically significant association with mortality. We hypothesize that extended LOS in patients with anxiety may be due to difficulty with oxygenation modalities (e.g., bilevel positive airway pressure (BIPAP) and continuous positive airway pressure (CPAP)) that require a facemask and can often exacerbate anxiety. Little research has been published on the effects of anxiety in BIPAP and appropriate sedation management [29]. We believe our findings set the stage for further research into oxygenation mechanisms in these anxiety groups to improve outcomes. Sedative abuse also increased LOS. The etiology of these findings requires further study.

Although we have a more uniform dataset than several previous studies, there were several limitations in this study. Our population of interest was different from prior work in this area. Applying our population’s findings, which included a more uniform wealth distribution, a different age distribution, and different comorbidities, to a population that looks more like the US may result in inappropriate conclusions and triaging of patients. It is unclear why the patient demographics differ. It may be related to the locations of the hospitals. Also, given the early nature of our data collection timeframe, some hospitals may not have yet created a robust testing infrastructure, and some patient subtypes may not have been included. Additionally, other SDoH and confounding variables are not included in our data, and variations in location may confound our use of insurance status to reflect socioeconomic status. We did not have vital signs and body mass index data available. Finally, our data do not include patients who may have died outside of the hospital. Patients without access to health care were likely not included in our population. The inadvertent exclusion of these patients probably kept some extremely sick and low-resource patients out of our analysis. Future researchers may wish to examine the influence of similar SDoH predictors and comorbidities after the introduction of vaccines and determine differences in outcomes.

## Conclusions

Using the large HCA Healthcare cohort, we found that comorbidities and insurance type were better predictors than race for predicting LOS and in-hospital mortality early in the COVID-19 pandemic. These findings support the contention that in COVID-19 infection, racial differences are most likely the result of differences in age, comorbidities, and socio-economic status and give a set of predictive variables that can be reviewed in future pandemics. Furthermore, our findings indicate that a stronger emphasis should be placed on access to care and managing comorbid factors to improve outcomes in future pandemics. More research should be done on the interactions between social determinants of health and outcomes to shape future public health policy.
